# Apolipoprotein M: Structural insights, functional roles, and therapeutic approaches in vascular disease

**DOI:** 10.1016/j.jbc.2026.111335

**Published:** 2026-02-28

**Authors:** Tim F. Dorweiler, Timothy Hla

**Affiliations:** Vascular Biology Program, Department of Surgery, Boston Children's Hospital, Harvard Medical School, Boston, Massachusetts, USA

**Keywords:** apolipoprotein M, ApoM, sphingosine-1-phosphate, S1P, S1P receptor, vascular, cardiometabolic, endothelium, ApoM-Fc, therapeutic

## Abstract

Apolipoprotein M (ApoM) is a lipocalin predominantly associated with high-density lipoprotein (HDL) that transports sphingosine-1-phosphate (S1P) in circulation. Through its stable binding and selective delivery of S1P to the endothelial S1P receptors (S1PRs), ApoM orchestrates a spectrum of vasoprotective effects. This review summarizes the structural characteristics of ApoM and its unique function as a sphingolipid chaperone, focusing on its role in vascular biology, specifically endothelial barrier integrity, vascular tone, and inflammation. We examine the biased signaling of ApoM–HDL–S1P through S1PR1 and its implications in modulating nitric oxide production and endothelial adherens junction assembly. In addition, circulating ApoM^+^–HDL appears to be important in transendothelial HDL transport and cholesterol efflux. Clinical and preclinical studies have linked reduced ApoM expression with cardiometabolic diseases, such as obesity, insulin resistance, type 2 diabetes, and chronic kidney disease, while emerging evidence also implicates ApoM in neurovascular, inflammatory, and retinal disorders. ApoM expression and plasma levels are regulated by hepatocyte nuclear factors, Forkhead box O nuclear transcription factors and inflammatory cytokines but also pharmacologically by statins and SGLT2 inhibitors. Recent development of engineered ApoM-based biologics, such as ApoM-Fc and ApoA1-ApoM fusion proteins, shows promise in preclinical models of vascular disease, demonstrating improvements in endothelial function, inflammation, and pathological neovascularization without inducing immunosuppression or bradycardia. Collectively, these insights position ApoM as both a critical biomarker and a therapeutic target for vascular health. Advancing ApoM-based therapies may offer a novel precision medicine strategy to treat cardiovascular and metabolic diseases through endothelial-targeted modulation of S1P signaling.

Cardiovascular diseases continue to be the leading cause of mortality in modern societies because of a confluence of lifestyle, medical, demographic, and environmental factors. Consequently, the implementation of effective prevention and management strategies is paramount in mitigating this burden (1). Sphingolipids are a diverse class of lipids built on a sphingoid (amino-alcohol) backbone, such as sphingosine, giving rise to more complex lipid species like ceramides, sphingomyelin, glycosphingolipids, and sphingosine-1-phosphate (S1P) that serve structural and signaling roles in cells. Sphingolipids have garnered significant attention in cardiovascular research in part because of the elucidation of the physiological and pathological actions of S1P and the plasma biomarker studies of ceramides and sphingomyelins that show strong associations of these lipids with atherosclerotic vascular disease (2, 3, 4, 5). Clinical studies have demonstrated remarkable correlations between specific circulating sphingolipids and the incidence and prevalence of cardiovascular adverse events (2, 3, 4, 6). However, the causal relationships of these associations remain uncertain since sphingolipids are structurally very diverse (>100,000 possible species), metabolically entangled with various other lipid pathways (*i.e.*, cholesterol and triglycerides), and technically challenging to quantify comprehensively (limited to approximately 400 sphingolipids *via* LC–MS/MS–based lipidomics) (7, 8).

Sphingolipid research has been focused on biosynthetic enzymes and receptor signaling, in contrast to upstream components, such as sphingolipid chaperones, which are defined as binding proteins, which stabilize, transport, and influence signaling functions. We first proposed this concept upon our discovery of ApoM as the key physiological chaperone of S1P that impacts its receptor-dependent functions (9, 10, 11). Recent evidence revealed how sphingolipid chaperones determine the physiological and pathological outcomes of downstream signaling events and biological outcomes. Apolipoprotein M (ApoM), a sphingolipid chaperone specific for S1P, was discovered in 1999 (12). Apolipoproteins are a diverse family of lipid-binding proteins that serve as the structural framework of lipoprotein particles. They facilitate reverse cholesterol transport (ApoA1 in high-density lipoprotein [HDL]), the delivery of atherogenic lipoproteins (ApoB in low-density lipoprotein [LDL]/VLDL), and specialized lipid transport associated with vascular calcification and thrombosis (Apo(a) in Lp(a)). Dysregulation of these apolipoproteins significantly predicts the development of atherosclerosis, coronary artery disease, peripheral artery disease, and aortic stenosis by promoting endothelial inflammation, plaque formation, and impaired fibrinolysis. Both circulating ApoM and S1P levels are inversely correlated with the onset and severity of cardiometabolic disease in clinical cohorts, unraveling the first sphingolipid-specific apolipoprotein with potential major significance in cardiometabolic diseases. This discovery has sparked interest in whether these associations could be causal and consequently therapeutically exploited (13, 14). This review highlights the structural and biochemical aspects of ApoM while exploring its therapeutic potential in cardiovascular and cardiometabolic diseases. By integrating molecular insights with translational approaches, this review aims to bridge current knowledge gaps and stimulate innovative strategies targeting endothelial dysfunction and vascular health for the prevention and/or treatment of cardiovascular diseases.

## ApoM structural insights

Circulating lipid chaperones are plasma proteins that bind and transport bioactive lipids, including S1P, lysophosphatidic acid, and other lipid mediators. Other intracellular lipid chaperones safeguard them from degradation, regulating their bioavailability, and facilitating proper metabolic channeling and signaling. The term “chaperone” underscores their dual function: protecting labile lipid mediators from enzymatic breakdown, preventing nonspecific interactions, and guiding lipid delivery in a spatially and temporally controlled manner to specific cells and receptors (15). Apolipoproteins play a crucial role as lipid chaperones by regulating metabolism, transport, clearance, and signaling. Dysregulation of apolipoproteins has been linked to dyslipidemia and an increased incidence and prevalence of cardiovascular disease. Specific apolipoproteins, including ApoB and ApoA-I, serve as key markers and mediators of vascular health. Elevated levels of ApoB are associated with arterial plaque formation and heightened cardiovascular risk, whereas higher levels of ApoA-I and HDL-associated apolipoproteins provide vasoprotective effects (16). Initially, the focus of lipoprotein investigations centered around cholesterol and triglycerides, but in the recent work, as exemplified by the discovery of ApoM as a carrier of the bioactive lipid S1P, researchers focused on the role and significance of apolipoproteins in the metabolism of signaling lipids.

**Figure 1:**
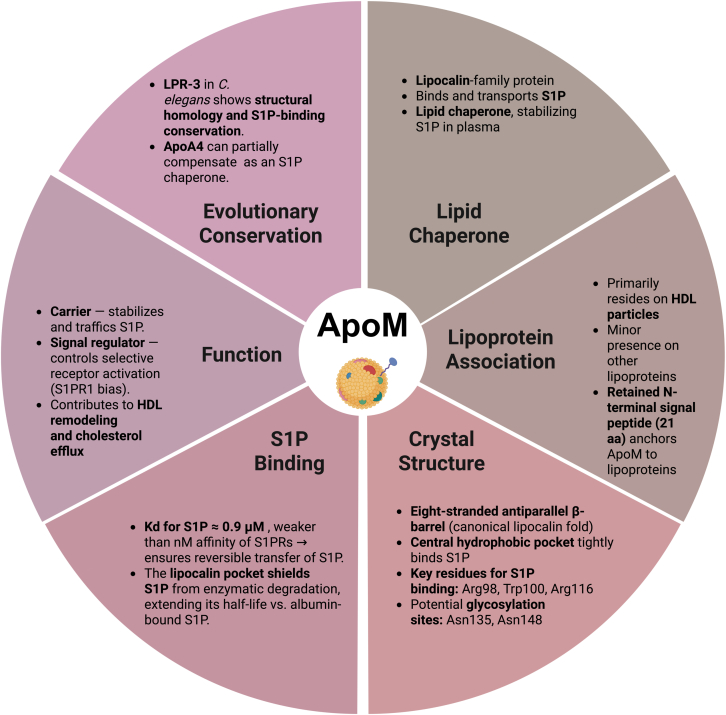
**Summary of structural properties of apolipoprotein M**.

ApoM is a 26 kDa protein belonging to the lipocalin family, which primarily associates with HDL particles in plasma, with minor associations with LDL and triglyceride-rich lipoprotein particles (Fig. 1). The *APOM* gene is located on chromosome 6p21.3 in humans, within the major histocompatibility complex class III region, spanning approximately 2.3 kbp and containing six exons and five introns. It encodes ApoM, which is produced and secreted by the liver and kidneys, with a minor contribution from adipocytes and enterocytes (17, 18, 19). Unlike other secreted proteins in which secretory signal peptides are cleaved in the mature form, ApoM retains the N-terminal signal peptide of 21 amino acids that is essential to anchor to lipoprotein particles (12). A 1.7Å resolution crystal structure of ApoM revealed an eight-stranded antiparallel β-barrel, forming a central hydrophobic lipocalin ligand-binding pocket allowing ApoM to bind and transport S1P, comprising 65% of its plasma pool (20). The dissociation constant (*K*_*d*_) of S1P to ApoM is approximately 0.9 to 0.95 μM for both human and murine ApoM (10), which is significantly lower than the nanomolar *K*_*d*_ of S1P to its receptors. While the primary function of ApoM is S1P trafficking, its structural contribution to HDL biology and lipoprotein remodeling has also been described (21, 22, 23). The broad role of ApoM in serum lipid homeostasis was further emphasized by gene polymorphisms (rs707921 and rs707922) being associated with variations in a variety of serum lipid parameters (triglyceride, HDL-cholesterol, and ApoB, *etc.*), with effects differing by ethnicity and gender (24).

S1P plays a crucial role in maintaining vascular health by regulating angiogenesis, vascular maturation, and barrier function through S1P receptors (S1PRs) on the endothelium. In addition, it influences immune cell trafficking and, consequently, inflammation *via* S1PRs on lymphocytes (11, 25, 26). Arg98, Arg116, and Trp100 residues within ApoM are essential for S1P binding, whereas the effects of other potential post-translational modifications (*i.e.*, glycosylation at Asn-135 and Asn-148) remain to be elucidated (27, 28). Of note, S1P chaperones seem to be evolutionary well conserved, in invertebrates and mammals, with multiple S1P chaperones having evolved to support complex and essential S1P signaling functions in a redundant manner. Lipocalin-related protein 3 exhibited remarkable similarities to mammalian ApoM in *Caenorhabditis elegans*, demonstrating high specificity in binding S1P under stress conditions. *In silico* calculations conducted using AlphaFold further revealed potentially significant structural similarities between lipocalin-related protein 3 and ApoM (29). Another study characterized compensatory S1P chaperones in ApoM and albumin double knockout mice. ApoA4 was discovered to partially retain circulating S1P levels, exhibiting S1P-binding properties. ApoA4-bound S1P was capable of activating S1PR1–3 and triggering intracellular signaling events, including ERK1/2 and Akt activation, even at nanomolar concentrations of S1P. ApoA4–S1P further enhanced vascular barrier function to the same extent as ApoM– or albumin–S1P, underscoring the significance of ApoA4's role in situations where ApoM or albumin is absent (30).

## ApoM and S1P

S1P is a bioactive circulating sphingolipid, synthesized *via* sphingosine kinase (SPHK) 1 in erythrocytes in mammals. Minor contributions of S1P synthesis originate from platelets and endothelial cells (10, 31, 32). Due to its polar phosphate moiety, S1P requires transporters to enter the circulation. Two transporters are currently known, MFSD2B in erythrocytes and platelets and spinster homolog 2 in the endothelium, contributing 50% and <10% of S1P levels in the blood, respectively (33, 34, 35). S1P acts *via* five G protein–coupled receptors (GPCRs), S1PR 1 to 5, orchestrating a broad plethora of downstream signaling and biological processes comprising cell migration, survival, fate determination, and gene expression (5, 8, 36). S1PR1–3 are expressed across various organs, tissues, and cells, with high expression patterns amongst the endothelium and immune cells, underscoring their significant implications for cardiovascular health and disease (37). S1PR4 is primarily expressed in immune cells and hence exerts limited effects on the cardiovascular system (38, 39). S1PR5 is expressed on some vascular cells and lymphocytes as well as in the central nervous system (CNS) and cancer cells. The effects of S1PR5 on the vasculature are less characterized and likely not as prominent given its low expression in vascular cells (40, 41, 42, 43, 44, 45). Interested readers are advised to consult recent reviews on S1P biology for a detailed discussion of this area of research (5).

Free S1P is rarely found but rather is associated with the circulating chaperone ApoM on HDL particles or albumin (10). The properties of S1P signaling, including its turnover, receptor engagement, and biological impact, are highly dependent on the nature of its carrier. ApoM-HDL binds S1P with higher affinity than albumin; presumably protecting the lipid in the lipocalin pocket with an unbinding energy of >60 kJ/mol for efficient release (12). It is hypothesized that receptor interaction may be required for the release of S1P from ApoM (46). In contrast, S1P binding sites on albumin are on the surface of this plasma protein and are estimated to have a lower energetic barrier of 20 kJ/mol, allowing faster dissociation and transient S1PR signaling (47). In addition, circulating S1P when bound to albumin is quickly recycled with a *t*_1/2_ of 15 min *via* dephosphorylation at the hepatocyte surface. The resulting sphingosine is then sequestered by SphK-2 phosphorylation and in turn degraded by intracellular S1P lyase (31, 48, 49, 50). It is hypothesized that circulating ApoM–S1P has a significantly longer *t*_1/2_ compared with albumin–S1P because of its protective lipocalin pocket. Swendeman *et al.* (51) reported that ApoM supplementation *in vivo* for 24 h increased plasma S1P and dihydro-S1P concentrations by 76.3 ± 13.7% and 52.9 ± 12.9%, respectively, in ApoM-deficient mice and by 29.9 ± 10.1% and 38.9 ± 4.1%, respectively, in wildtype mice, with other major circulating lipids being unaffected. These data suggest that ApoM stabilizes bound S1P as a soluble protein *in vivo*, presumably by protecting it from phosphatase-mediated degradation. Together, kinetic and structural differences lead to distinct receptor activation profiles and downstream signaling consequences (Table 1).

**Table 1:** Differences in key features of albumin–S1P and ApoM–HDL–S1P

Feature	Albumin–S1P	ApoM–HDL–S1P
S1PR1 binding affinity (10)	Lower, S1P readily released	High, S1P tightly bound
S1PR1 signal duration (151)	Short lived, transient	Sustained, prolonged
S1PR1 receptor internalization (11)	Rapid, strong decrease in S1PR	Sustained, limited decrease in S1PR
S1PR1-mediated endothelial barrier enhancement (11, 152)	Short lived	Prolonged
S1PR pathway selectivity (11, 51, 60)	Activates all S1PRs, unbiased	More selective for S1PR1, biased

Upon release from the carrier protein, S1P binds to S1PRs, triggering G-protein signaling. G-protein signaling is a cell communication process in which ligand-activated GPCRs catalyze GDP–GTP exchange on heterotrimeric (made of three different subunits) G proteins, consequently leading the Gα-GTP and Gβγ subunits to regulate downstream enzymes or ion channels until GTP hydrolysis terminates the signal. Each receptor is coupled to specific G protein families, with S1PR1 exclusively being coupled to Gαi/o, whereas S1PR2 and S1PR3 are more promiscuous and can couple to Gαi/o, Gαq/11, and Gα12/13. S1PR4 and S1PR5 bind to Gαi/o, Gα12/13, with some studies also suggesting Gαq as a potential signaling pathway. However, S1PR2 and S1PR3 show a preference for Gα12/13 and Gαq/11, respectively (52, 53, 54). As such, cells that are endowed with at least one or more S1PR subtypes can mediate diverse downstream effects in response to receptor activation. Subsequent to G protein activation, GPCR kinase–dependent GPCR phosphorylation followed by ß-arrestin recruitment suppresses G-protein activation and terminates the signal.

S1PR1, the first S1PR identified, is the most extensively studied among the five GPCRs (25). Clinically approved S1PR1 modulators, such as fingolimod (FTY-720), have been utilized in the treatment of multiple sclerosis since 2010. FTY-720, once phosphorylated by SphK-2, is a broad specificity S1PR agonist, activating S1PR1, 3, 4, and 5 with low-nanomolar affinity but does not activate S1PR2 (55). FTY-720-P induces β-arrestin–dependent receptor internalization, linking the receptor to clathrin-coated pits, forming clathrin-coated vesicles, which traffic to late endosomes and lysosomes for degradation in an Rab9-dependent manner (56, 57, 58, 59). In multiple cell types, the irreversibility of S1PR1 modulator–induced receptor internalization enables receptor degradation while minimizing recycling back to the cell surface. This ultimately inhibits the egress of lymphocytes from secondary lymphoid organs and thereby mitigates the autoimmune inflammatory response at the target organs (56). While effective, many of FTY720's beneficial effects can be compromised by the lack of receptor specificity, sparking interest in the development of more receptor-specific small molecules (55). In addition to the S1PR1 critical function in lymphocyte trafficking, its role as a central regulator of vascular health has been well characterized.

Endothelial ApoM–HDL–S1PR1 signaling is a key pathway modulating vascular tone *via* nitric oxide (NO). ApoM–HDL-bound S1P activates S1PR1 on the endothelial cell surface with sustained kinetics, exhibiting selectivity for the receptor that interacts with Gαi/o exclusively and consequently initiates the release of Gβγ subunits. Initially, downstream Gβγ subunits activate PI3K, which phosphorylates PIP2 to PIP3. PIP3 then recruits Akt (protein kinase B), which is subsequently phosphorylated, activating endothelial NO synthase, stimulating NO production (60, 61). NO exerts vasoprotective effects primarily by promoting vasodilation *via* soluble guanylate cyclase in the vascular smooth muscle to maintain blood flow, inhibiting platelet aggregation to prevent thrombosis, suppressing vascular smooth muscle cell proliferation to avoid pathological remodeling, and reducing leukocyte adhesion and inflammation to protect endothelial integrity (10). The multifaceted actions of NO collectively preserve vascular homeostasis and prevent endothelial dysfunction associated with cardiovascular pathology (62, 63, 64). Successively to ApoM–HDL–S1PR1 pivotal role in endothelial NO synthesis, evidence emerged demonstrating endothelial junction assembly and tightening as a secondary mechanism that contributes to vascular preservation.

Lee *et al.* first discovered S1P's pivotal role in vascular junction architecture by activating S1PR1 and S1PR3 on endothelial cells. S1P–S1PR1/3 signaling triggers both the Gi/mitogen-activated protein kinase/cell survival pathways and the Rho/Rac-dependent assembly of adherens junctions, namely vascular endothelial (VE)-cadherin, which are crucial for maintaining the integrity of the endothelial barrier. The study demonstrated that S1P synergizes with conventional angiogenic growth factors (VE growth factor and fibroblast growth factor) to facilitate the formation of mature and stable capillary networks both *in vitro* and *in vivo*. These findings position S1P as a key signaling molecule in the regulation of endothelial cell junctions and vascular network formation, exerting its effects through specific receptor-mediated pathways (65).

Burg *et al.* complemented the importance of S1P–S1PR1 in vascular health by reporting that endothelial cell S1PR1 is essential for preserving vascular junction biology during inflammatory arthritis. Employing mouse models of serum-induced arthritis, loss or pharmacological inhibition of endothelial S1PR1 resulted in enhanced vascular leakage and more severe joint inflammation, whereas S1PR1 overexpression or agonism mitigated these effects. Mechanistically, S1PR1 signaling restricted the cleavage (shedding) of VE-cadherin by inhibiting the activity of the metalloproteinase ADAM10. Consequently, endothelial junctions were preserved, and vascular permeability was reduced. Notably, patients with active rheumatoid arthritis exhibited diminished circulating S1P and reduced S1PR1 expression in microvessels, indicating that disruption of this axis contributes to disease pathology. Therefore, S1PR1 emerges as a promising therapeutic target in inflammatory vascular diseases (66).

Recently, a study by Janiurek built on the work of Yanagida *et al.* (67) and further emphasized the role of HDL–ApoM–S1PR1 in microvascular beds of the CNS, investigating this signaling pathway in permeability regulation of the blood–brain barrier (BBB). Employing two-photon microscopy in ApoM-knockout mice, the study observed a significant enhancement in paracellular BBB permeability to small molecules (1–5 kDa), despite the absence of apparent structural alterations in endothelial junctions. The absence of ApoM–S1P also resulted in increased vesicle-mediated (transcytotic) transfer of albumin across arteriolar endothelium, but not in capillaries or venules, indicating a vessel-type–specific effect. Administration of the S1PR1 agonist SEW2871 promptly restored BBB integrity in ApoM-knockout mice, underscoring the pivotal role of the ApoM–S1P–S1PR1 axis in preserving both paracellular and transcellular BBBs. Consequently, targeting this pathway may offer protective mechanisms against BBB dysfunction in neurological disorders (68).

Beyond barrier maintenance, ApoM–S1P–S1PR1 signaling plays a central role in transendothelial HDL transport, as shown in both murine and human studies. HDL isolated from humans lacking ApoM or ApoM-deficient mice exhibited significantly diminished binding, association, and transport across endothelial cells compared with ApoM-containing HDL. Pharmacological activation of S1PR1 enhanced HDL uptake and transport, whereas S1PR1 inhibition attenuated these processes. Notably, silencing scavenger receptor class B type I rendered S1PR1-mediated HDL transport stimulation ineffective. *In vivo*, endothelial-specific S1PR1 expression increased HDL transport from blood to the peritoneal cavity and upregulated aortic silencing scavenger receptor class B type I expression, concomitantly reducing albumin transport. These findings define a unique role for ApoM–S1P–S1PR1 in facilitating selective, active HDL transport, distinct from passive filtration mechanisms (69).

**Figure 2:**
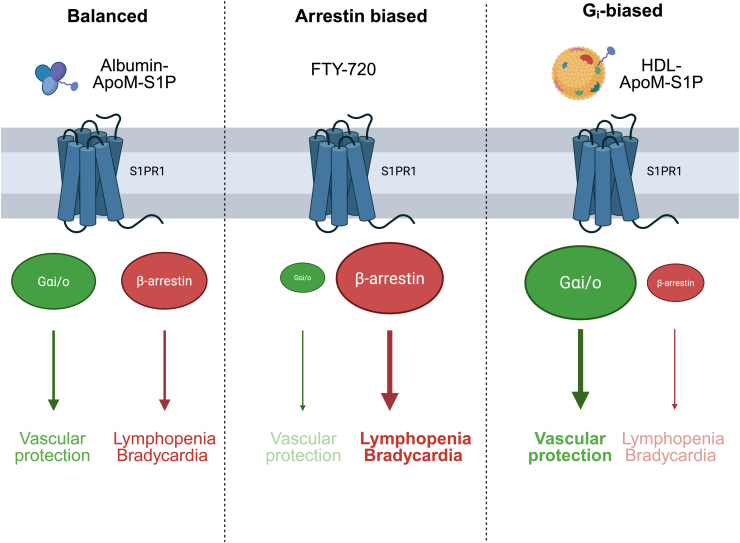
**G-protein signaling mechanisms of albumin–S1P, FTY-720, and ApoM–HDL–S1P.** ApoM, apolipoprotein M; HDL, high-density lipoprotein; S1P, sphingosine-1-phosphate.

G protein–biased signaling refers to functional selectivity observed at GPCRs. In this phenomenon, a ligand binds to a GPCR and selectively activates a specific G protein–dependent signaling pathway over β-arrestin pathways at the same receptor. Consequently, the receptor's response is biased toward Gi-mediated effects, including inhibition of adenylyl cyclase and reduction of cAMP levels. This bias is particularly significant in the case of HDL, as it impacts intracellular pathways related to cardiovascular physiology or lipid metabolism. The selective coupling of GPCRs to Gi protein signaling pathways can be attributed to the fact that different ligands can stabilize distinct receptor conformations, favoring binding to certain intracellular signaling proteins (such as G proteins) over others (Fig. 2) (70). HDL–ApoM–S1P–S1PR1 shows Gi-biased signaling over ß-arrestin–dependent GPCR internalization on endothelial cells is crucial to robustly maintain vascular barrier integrity and suppress pathological angiogenesis (11, 51), whereas other S1PRs, such as S1PR2, may exert opposing, detrimental effects by promoting vascular permeability and disrupting endothelial junctions. Albumin-bound S1P dissociates more readily because of its lower binding affinity and higher dissociation rate, leading to broader and more transient activation of multiple S1PRs, including S1PR2. In a study conducted by Sanchez *et al.*, the researchers investigated the differential regulation of endothelial barrier function by S1PR subtypes with a specific focus on S1PR2. Their study revealed that selective activation of S1PR2 in endothelial cells triggered Rho–Rho-associated coiled-coil containing protein kinase and phosphatase and tensin homolog–dependent signaling pathways. This activation resulted in the disruption of adherens junctions, specifically VE-cadherin, the formation of increased actin stress fibers, and an elevation of paracellular permeability. Furthermore, pharmacological inhibition of S1PR2 with JTE013 demonstrated enhanced barrier-stabilizing effects of S1PR1, including cortical actin formation and adherens junction assembly, both *in vitro* and in a rat lung model of vascular permeability (71). In the retinopathy of prematurity model, Skoura *et al.* (72) showed that S1PR1 induction is required for driving retinal vascular leakage and abnormal angiogenesis. Kim *et al.* emphasized the pivotal role of S1PR2s as a pathological mediator of vascular permeability and injury in an experimental model of stroke and CNS injury. Genetic deletion or pharmacological inhibition of S1PR2 in mice resulted in diminished BBB disruption, intracerebral hemorrhage, and neurovascular injury following ischemic stroke. Mechanistically, S1PR2 signaling enhanced the activity of matrix metalloproteinase-9 and gelatinase in cerebral microvessels, contributing to vascular breakdown. Notably, S1PR2 expression was specifically upregulated in ischemic brain endothelium in both mice and humans (73). Collectively, these studies conclude that S1PR2 operates in a counteractive manner to S1PR1, promoting vascular leak. Consequently, the equilibrium between these receptors determines endothelial barrier integrity in the CNS, suggesting that a combination of ApoM–S1P–S1PR1 agonism and S1PR2 antagonism could potentially be therapeutic in conditions such as pulmonary edema and sepsis.

Finally, these reports further underscore that targeting S1PR1 with high specificity is indispensable to harness the vascular protective effects of S1P while avoiding the barrier-disrupting actions mediated by other S1PR subtypes.

## ApoM regulatory mechanisms

The majority of circulating ApoM is synthesized by the liver and kidneys, and thus, extensive research is being conducted on the regulation of expression in these tissues. Due to the very limited capacity of adipocytes and enterocytes in the synthesis of ApoM, this area remains understudied, and the mechanisms regulating adipocyte-derived ApoM remain largely unknown.

Hepatic and renal-specific expression of *Apom* is regulated by an interplay of not only transcription factors and nuclear receptors but also inflammatory mediators (74). Hepatocyte nuclear factors (HNFs) are transcription factors that are extensively dysregulated in and, on occasion, even causative of cardiometabolic diseases. HNFs bind to regulatory elements in the *Apom* promoter region (position −33 to −21 relative to the transcription start site) and are hence present as central regulators of *Apom* transcriptional regulation, further emphasizing the connection of ApoM to cardiometabolic disease. HNF-4α is a primary activator of *Apom* transcription in the liver and kidney *via* a conserved binding motif in its promoter. Mutations in HNF-4α are causal of maturity-onset diabetes of the young type 3 and lead to profoundly reduced *APOM* expression. Thus, circulating ApoM levels have been proposed for diagnostic purposes, allowing for discrimination of HNF-4α -MODY from type 1 diabetes (75). Liver receptor homolog-1 (LRH-1) also directly regulates *Apom* transcription by binding to its promoter. Interestingly, bile acids suppress LRH-1 activity and binding to the *Apom* promoter through a small heterodimer partner–dependent mechanism (76). Through binding to the hormone response element of ApoM in its promoter, HNF-4α further interacts with other nuclear receptors, such as retinoid X receptor, retinoic acid receptor, thyroid hormone receptor, peroxisome proliferator–activated receptor, and liver X receptor, postulating a complex regulatory network underlying hepatic *Apom* expression regulation (77).

The importance of the hormone response element for *Apom* is accentuated as estrogen receptor α can bind to 17β-estradiol, subsequently binding to similar motifs in the *ApoM* promoter region and thus enhancing its expression. The upregulation of *ApoM* expression *via* estrogen receptor α binding suggests hormonal regulation and should be further explored (78).

The position of ApoM within the major histocompatibility complex class III genomic region facilitates its proximity to genes implicated in immune and inflammatory responses, suggesting a potential role for inflammatory signaling pathways in regulating its expression. In contrast to HNF's stimulating role, inflammation-associated transcription factors repress *Apom* expression. AP-1 transcription factors, namely c-Jun and JunB, have been shown to be upregulated under proinflammatory conditions and to compete with HNF-1α for binding to the promoter, leading to repression of expression (79). Early evidence further suggests that bacterial and viral infections decrease *Apom* mRNA and circulating protein levels in humans, which was supported by *in vitro* hepatocyte experiments using lipopolysaccharide (LPS), tumor necrosis factor-α, or interleukin 1, showing reduced *Apom* expression (80).

With accumulating evidence emerging that ApoM levels change under metabolic dysfunction, studies have investigated endocrine hormonal regulation and how therapeutic interventions affect *ApoM* expression regulation. Transcription factors of the Forkhead box (FOX) family are essential regulators of hepatic gene expression, controlling, amongst others, glucose homeostasis in response to glucagon and insulin (81). FOXOs have been found to be sensitive to metabolic dysfunction, with strong dysregulation observed in diabetes (82). FOXO transcription factors were found to enhance ApoM expression by directly binding to its promoter and acting as positive regulators of its transcription. Loss of FOXO activity in L-FoxO1,3,4-deficient mice reduced ApoM levels, highlighting their critical role in lipoprotein dysfunction observed amongst metabolic diseases (83). The PI3K pathway is crucial for insulin signaling in the human body and cardiovascular health and function. In cardiometabolic diseases, this pathway is widely dysregulated. In HepG2 cells, ApoM expression is repressed *via* PI3K signaling, when insulin and insulin-like growth factor I are present in excess (84). Statins target hypercholesteremia in patients affected or at risk of developing dyslipidemia, diabetes mellitus, and cardiovascular diseases. The lipophilic statin atorvastatin increased *ApoM* expression in hyperlipidemic models by attenuating LXRα expression, suggesting that established therapeutic avenues in cardiometabolic diseases can modulate ApoM levels (85).

Reduced *ApoM* expression, driven by genetic mutations, inflammation, or metabolic disturbances, can exacerbate key features in the progression of cardiometabolic diseases. Therefore, maintaining or enhancing *ApoM* expression through lifestyle interventions or pharmacological means may offer therapeutic potential in preventing or mitigating vascular, inflammatory, and metabolic disease progression (Table 2).

**Table 2:** Summary of how endocrine, metabolic, and inflammatory cues tightly regulate ApoM transcription

Regulator type	Key factor(s)	*Apom* effect	Mechanism	Comments
HNFs (75, 76)	HNF-1α, HNF-4α	↑	Bind conserved promoter motifs (−33 to −21); activate ApoM transcription	HNF-4α mutations → ↓ ApoM (MODY3) → possible diagnostic biomarker
Nuclear receptors (74, 77)	LRH-1, retinoid X receptor, retinoic acid receptor, thyroid hormone receptor, peroxisome proliferator–activated receptor, liver X receptor	↓	Form heterodimeric complexes with HNF-4α; integrate lipid and hormone signaling	Bile acids suppress LRH-1 *via* a small heterodimer partner → ↓ ApoM
Hormonal regulation (78)	Estrogen receptor α (ER-α)	↑	17β-estradiol → ER-α → binds hormone response element → ↑ ApoM	Suggests sex-dependent control
Metabolic sensors (83)	FOXO1/3/4	↓	Bind ApoM promoter, enhance transcription; loss → ↓ ApoM	Link between insulin resistance and ApoM downregulation
Inflammatory repressors (79)	AP-1 (c-Jun, JunB)	↓	Compete with HNF-1α for promoter → ↓ ApoM under inflammation	LPS, tumor necrosis factor-α, interleukin-1 all reduce ApoM in hepatocytes
Pharmacological modulators (85)	Statins, SGLT2 inhibitors	↑	Statins (atorvastatin) ↑ ApoM *via* LXRα modulation; SGLT2i (dapagliflozin) preserve ApoM under stress	Therapeutic upregulation of ApoM *via* lipid/glucose drugs

## ApoM clinical and preclinical evidence

**Figure 3:**
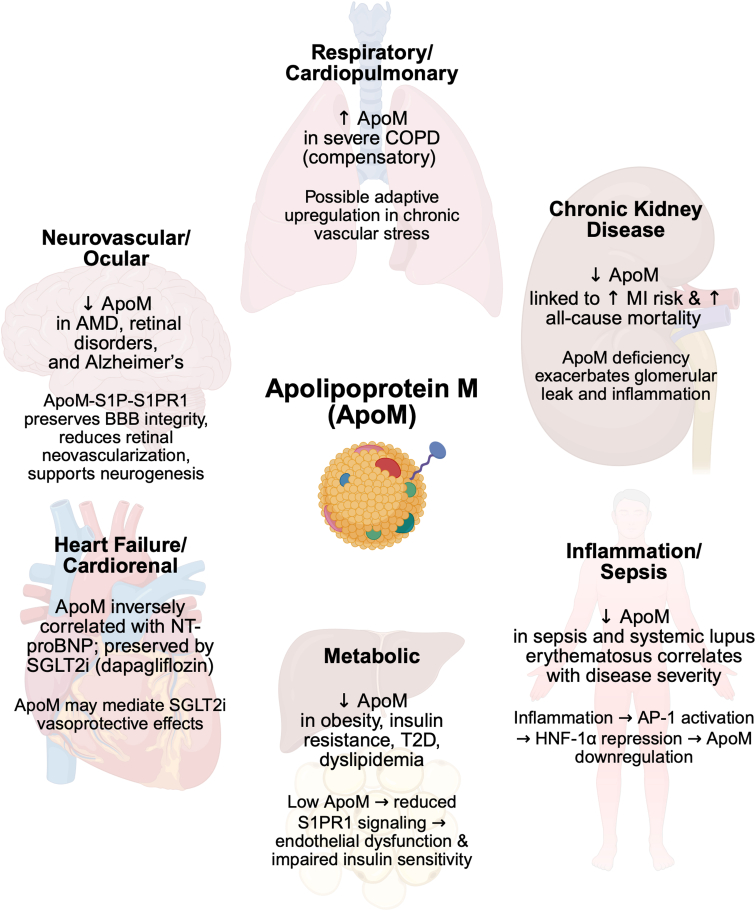
**Summary oftranslational andpreclinicalevidence of ApoMassociations and effects.** ApoM, apolipoprotein M.

ApoM, as part of the ApoM–HDL–S1P complex, exerts vasoprotective effects by maintaining endothelial integrity, reducing inflammation, and mediating antiatherogenic signaling *via* S1PR1 on endothelial cells (5, 8). These observations have prompted extensive investigations into how circulating ApoM levels are associated with various disease states across cardiovascular, metabolic, inflammatory, neurodegenerative, ocular, renal, and lipid-related disorders, all of which share pathophysiologic links to vascular health and disease progression (Fig. 3).

Metabolic syndromes and dyslipidemias are closely tied to macrovascular and microvascular complications, representing key risk factors for major adverse cardiovascular events. Circulating ApoM levels have emerged as important biomarkers and potential modulators of insulin sensitivity, lipid metabolism, and cardiovascular outcomes. Multiple studies consistently show that lower ApoM levels correlate with increased cardiometabolic burden, including obesity, insulin resistance, dyslipidemia, and type 2 diabetes. For instance, individuals with type 2 diabetes but without hyperlipidemia exhibited increased circulating ApoM levels (22.61 ± 10.81 ng/μl *versus* 26.63 ± 10.35 ng/μl), whereas ApoM levels were lower in individuals with dyslipidemia (22.61 ± 10.81 ng/μl *versus* 18.54 ± 10.33 ng/μl) and hypertension (22.61 ± 10.81 ng/μl *versus* 19.83 ± 7.41 ng/μl) (86). Furthermore, ApoM exhibited negative associations with homeostatic model assessment of insulin resistance (HOMA-IR) and high-sensitivity C-reactive protein (CRP) (*r* < −0.2) and positive correlations with HDL markers (HDL-cholesterol and ApoA1, *r* > 0.3). Conversely, ApoM was favorably associated with LDL markers (LDL-C and ApoB100, *r* < 0.20) and adversely correlated with insulin and age (*r* < −0.2) (87). Notably, ApoM was the sole negative determinant of HOMA-IR in multiple regression models. Following a bariatric surgery slimming program, the change in HOMA-IR was solely negatively associated with the decrease in circulating ApoM (*r* = −0.71) (87). Reduced ApoM expression and/or protein levels in metabolic diseases were confirmed *in vivo* by employing diet-induced obesity and *Lepr*^*db/db*^ genetic models modeling insulin-resistant diabetes while chemically streptozotocin-induced insulin-dependent models of diabetes reported increased ApoM levels (83, 87, 88). This contradiction sparks interest in potential implications of streptozotocin influencing ApoM levels because of its off-target effects on hepatocytes, a pivotal site of ApoM production, or if the phenotype observed is due to the insulin-dependent nature of this model. Chronic kidney disease (CKD), the second most common and arguably most fatal complication of diabetes, is also associated with reduced ApoM levels. Clinically, circulating ApoM levels demonstrated a significant inverse association with the risk of myocardial infarction (MI) (hazard ratio [HR] = 0.60, 95% confidence interval [95% CI] = 0.49–0.75) and all-cause mortality (HR = 0.63, 95% CI = 0.48–0.83) in patients with CKD with heterogeneous ApoM–S1P plasma levels across CKD etiologies often linked to confounders like diabetes and atherosclerotic vascular disease (89, 90). This evidence on ApoM in CKD has been supported by an abundance of preclinical *in vivo* data, outlining ApoM supplementation as a potential novel therapeutic approach, as summarized previously by Christoffersen *et al.* (91)in detail. In short, experimental models show that ApoM-bound S1P preserves glomerular and peritubular microvascular integrity, mitigates proteinuria, and limits tubular apoptosis and interstitial inflammation through S1PR1-dependent enhancement of endothelial barrier function and anti-inflammatory signaling (10, 92). Complementary gain- and loss-of-function studies demonstrate that ApoM overexpression or pharmacological augmentation of the ApoM–S1P axis attenuates fibrosis and chronic injury in nephropathies, such as IgA nephropathy, whereas ApoM deficiency, impaired tubular reabsorption of ApoM (93, 94), or a shift toward S1PR2/3 signaling exacerbates ischemia–reperfusion damage, capillary rarefaction, and matrix accumulation (95, 96). This collectively supports ApoM/S1P-targeted augmentation as a kidney-protective strategy in CKD.

Heart failure with reduced ejection fraction is another indication closely related to metabolic, lipid, and also kidney pathology, affecting 3 million Americans in 2023 (97). In patients with heart failure with reduced ejection fraction (0.641 ± 0.181 mM) from the DEFINE-HF trial (NCT02653482), higher ApoM levels were associated with lower levels of the heart failure biomarker NT-proBNP (β = 0.11), particularly in those treated with the SGLT2 inhibitor dapagliflozin (β = 0.19, *p* < 0.001; *P* interaction = 0.025). This inverse relationship was most significant in individuals with albuminuria (elevated urinary albumin-to-creatinine ratio), suggesting that ApoM may mediate dapagliflozin's cardiorenal benefits in the context of kidney dysfunction (−0.28 per 0.1 mM increase in ApoM). Although dapagliflozin did not significantly alter overall ApoM levels over 12 weeks, its effects on cardiac biomarkers appear to be modulated by baseline kidney injury and changes in ApoM (98). In addition, SGLT2 inhibition with dapagliflozin preserved ApoM levels during acute inflammation in patients diagnosed with coronavirus disease 2019 (COVID-19) and a preclinical LPS-induced mouse model. Clinically, COVID-19 patients receiving dapagliflozin in the ACTIV-4a trial (NCT04505774) exhibited significantly higher circulating ApoM levels compared with those receiving standard care alone (0.5240 *versus* 0.6537 mM). In mice induced with LPS-induced inflammation, dapagliflozin pretreatment maintained ApoM levels (0.017 *versus* 0.035 a.u./ml) by preserving megalin (LRP2) receptor function, which reduced endothelial barrier leak and improved neutrophil behavior. These findings demonstrate a consistent correlation between SGLT2 inhibition and preserved ApoM levels across species, associated with enhanced vascular integrity during inflammatory stress and cardiac dysfunction (99).

Cardiopulmonary disorders and vascular diseases are intrinsically linked through shared risk factors, particularly inflammation. They exhibit common mechanisms, such as chronic systemic inflammation, and frequently overlap in clinical manifestations. Consequently, the simultaneous presence of both conditions leads to heightened morbidity and mortality (100). Clinical evidence further stresses this strong linkage by inversely correlating circulating ApoM with cardiopulmonary pathology and endothelial dysfunction. In chronic obstructive pulmonary disease, serum ApoM levels are elevated and increased with disease severity but were not associated with coronary artery disease (odds ratio [OR] = 1.095, 95% CI = 1.034–1.160). This increase was the most pronounced among measured proteins in severe cases (101). Anthracycline-induced cardiotoxicity induced by the chemotherapeutic drug doxorubicin offers a distinct example of protective potential by ApoM. In both patients and mouse models, higher ApoM levels mitigated cardiac damage by sustaining myocardial autophagic flux and preserving lysosomal integrity. Clinically, reduced circulating ApoM levels correlated with higher mortality in patients with anthracycline-induced heart failure, establishing an inverse association between ApoM and adverse outcomes (HR = 0.47, 95% CI = 0.25–0.88). This protective role was confirmed in animal models where ApoM heterozygosity increased doxorubicin-induced mortality in mice, whereas elevated ApoM expression mitigated cardiotoxicity and lysosomal damage (102).

Circulating ApoM levels have been extensively studied in peripheral vascular diseases but not in the vasculature of the CNS. Emerging evidence links ApoM to neurovascular health, particularly in diseases characterized by disrupted lipid metabolism and vascular barrier dysfunction. In age-related macular degeneration (AMD), people affected have significantly reduced circulating levels of ApoM, which is linked to impaired cholesterol metabolism and retinal damage. Investigations using UK Biobank data further confirmed a negative association between plasma levels of ApoM and the prevalence (OR = 0.6, 95% CI = 0.45–0.80) as well as incidence (OR = 0.75, 95% CI = 0.65–0.86) of disorders of the choroid and retina, including AMD (103). Mouse models of neovascular and dry AMD confirmed that restoring ApoM, either genetically or pharmacologically, reduces retinal cholesterol buildup, inflammation, and abnormal blood vessel growth, while improving retinal pigment epithelium function. Mechanistic studies reveal that ApoM–S1P activates protective signaling pathways in the retina *via* S1PR1 and S1PR3, further supporting the therapeutic potential of S1PR1 signaling. Together, these findings suggest that targeting ApoM could offer a novel strategy to slow or prevent AMD progression by correcting underlying lipid metabolism defects (104, 105). Similarly, in Alzheimer's disease, elevated plasma ApoM and altered lipid ratios are associated with disease presence and cognitive decline (OR = 1.058, 95% CI = 1.027–1.090), underscoring a broader role in neurodegenerative processes (106). A recent study reveals that circulating ApoM-bound S1P levels are significantly diminished in early Alzheimer's patients, correlating with olfactory impairment and ventricular enlargement as indicators of neurovascular dysfunction. In ApoM-deficient and APP/PS1 mouse models, this deficiency disrupts ependymal cell polarity, ciliary organization, cerebrospinal fluid flow, and subventricular zone neurogenesis, resulting in ventriculomegaly. ApoM–S1P preferentially accesses the subventricular zone through specialized vasculature, signaling through S1PR1 on neural stem and ependymal cells to maintain niche integrity. Transgenic ApoM overexpression or serum transfer fully rescues these phenotypes, confirming causality of reduced ApoM levels and neurological dysfunction, while also giving significant merit for ApoM replacement therapy in, for example, Alzheimer's patients. These findings extend the vascular-protective roles of ApoM–S1P to neurovascular niches, underscoring its therapeutic potential in vascular diseases (103).

Endothelial inflammation is a key driver of cardiovascular, metabolic, and neurodegenerative disorders (107). Circulating ApoM levels are highly responsive to both systemic and tissue-specific inflammation, typically decreasing in such contexts. Systemic lupus erythematosus (SLE) arises because of an autoimmune reaction causing systemic inflammation. Serum ApoM levels were found to be significantly lower in SLE compared with healthy controls and are negatively correlated with disease activity, as evidenced by negative correlations with SLEDAI and anti-dsDNA antibodies (*r* = −0.551, −0.562) (108). Sepsis presents another inflammatory context where ApoM levels drop precipitously. Decreased ApoM concentrations were observed in the plasma of patients affected by sepsis-related deaths (12.00 ± 3.13 mg/dl) compared with controls (26.50 ± 3.56 mg/dl), paralleling reductions in cholesterol and HDL in these individuals. Plasma ApoM levels decreased dramatically during sepsis, to about 60% of postrecovery levels, indicating a roughly 1.7-fold decrease (109).

IgA-mediated vasculitis is another inflammation-driven disease, formerly known as Henoch–Schönlein purpura, characterized by a systemic small vessel vasculitis with accumulation of IgA-containing immune deposits in predominantly dermal, gastrointestinal, joint, and renal microvessels. This deposition leads to inflammation, microvascular leakage, and the distinctive clinical manifestations associated with the condition. ApoM was found to be elevated in IgA vasculitis patients compared with healthy controls. Notably, ApoM levels were reduced in patients with nephritis compared with those without nephritis. ApoM levels were higher in classes I and II IgA vasculitis nephritis patients compared with classes III and IV. ApoM levels were further identified as a potential serum biomarker, with levels below 24.81 mg/l serving as an independent predictive factor for IgA vasculitis nephritis. This factor was independently associated with the presence of nephritis in IgA patients (OR = 0.32, 95% CI = 0.12–0.85). Serum ApoM concentration also inversely correlated with the *International Study of Kidney Disease in Children* scores in IgA vasculitis nephritis patients, suggesting a potential protective role in disease progression (110).

Mixed or complex disorders that exist at the inflammatory–metabolic interface with infections and/or autoimmune diseases are greatly influenced by the health of the vascular system. These conditions share features of chronic inflammation, endothelial barrier dysfunction, and metabolic dysregulation with pathologies described above (111). In COVID-19, decreased circulating ApoM levels are observed alongside increased CRP, and both are significantly associated with disease severity and adverse outcomes. ApoM further showed a significant negative correlation with clinical severity indices that also rise with CRP, suggesting an inverse relationship between inflammation measured as CRP and ApoM levels in acute illness (112). In animal models, results confirmed a causal relationship of chronic elevation of CRP leading to approximately a twofold increase in serum ApoM and other apolipoproteins. This indicates that persistent inflammation can upregulate ApoM expression, though this has yet to be directly confirmed in human clinical cohorts (113). Inflammatory bowel disease is characterized by chronic inflammation, comparable to phenotypes observed in metabolic diseases and comprises a variety of pathologies, such as ulcerative colitis (UC) and Crohn's disease (CD). Serum ApoM levels were significantly higher in UC (13.5 ± 8.8 μg/ml) and CD (10.2 ± 5.8 μg/ml) compared with healthy controls (2.0 ± 1.3 μg/ml), reflecting a roughly five- to sevenfold increase in UC and a four- to fivefold increase in CD.

**Figure 4:**
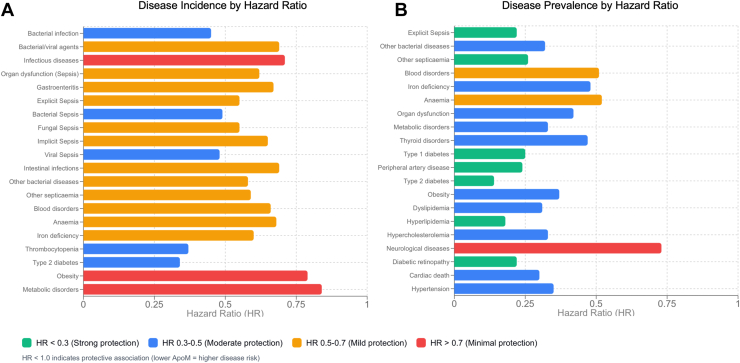
**Top 20disease incidence** (*A*) and prevalence (*B*) associated with circulating ApoM levels in the UK Biobank. ApoM, apolipoprotein M.

To provide a more comprehensive overview of clinically relevant associations that correlated with circulating ApoM levels, we synthesized key findings from the UK Biobank proteome-wide analyses with both prevalent and incident disease outcomes. The summarized evidence underscores diseases with the strongest correlations, ranging from cardiovascular, renal, ocular, and neurodegenerative domains, where ApoM consistently emerged as a potentially protective biomarker or risk modifier. The figure below presents the 20 most significant (*p* < 0.0001) incident events and prevalent disorders associated with ApoM levels, emphasizing its broad relevance across vascular-related pathologies (Fig. 4) (114).

## Therapeutic strategies

Cardiovascular diseases account for almost 18 million deaths annually, including over 900,000 in the United States alone, which present a giant urgent unmet need for novel vascular-protective therapies. Notably, disparities in premature vascular disease mortality across US communities underscore the importance of equitable and innovative interventions (115).

The crucial role of S1PR1 in vascular homeostasis makes the receptor a pivotal target for cardiovascular disease prevention and intervention. The FREEDOMS (NCT00086450), FREEDOM II (NCT03952039), and TRANSFORMS (NCT03575351) clinical trials investigated the safety and efficacy of the small-molecule S1PR1 agonist fingolimod (FTY-720) in multiple sclerosis and suggested that adverse effects occurred because of endothelial S1PR1 internalization. Adverse events included but were not limited to bradycardia at the first dose or atrioventricular block, macular edema, mild hypertension, rendering fingolimod unfit for cardiovascular intervention (116, 117, 118, 119). Highly selective and Gα_i_-biased S1PR1 agonism without receptor internalization could address clinical concerns and may present a potential approach to selectively target the endothelium and promote its health (see below). Thus, ApoM supplementation may present a crucial opportunity to develop a first-in-class modality that is specifically designed to promote vascular health *via* S1PR1 signaling with high specificity while avoiding receptor internalization. Supplementation of HDL particles containing ApoM is very challenging, with large-scale clinical trials consistently reporting a lack of clinical benefit despite raising HDL levels as well as a necessity for impractical repeated intravenous infusions for long-term therapy (*i.e*., NCT01352594) (120, 121, 122, 123). Notably, reconstituted HDL therapy using ApoA1 also failed to achieve clinical benefit, emphasizing the complex nature of HDL-based therapeutics (NCT00254137) (124). When not associated with HDL, free ApoM presents significant pharmacokinetic disadvantages as its half-life *in vivo* is shorter than 15 min (125). To address these pharmacokinetic concerns, researchers have employed first-generation fusion proteins with improved pharmacological properties, namely ApoM-Fc, and second-generation fusion proteins, such as ApoM-A1M, to enhance the therapeutic properties of ApoM while promoting S1PR1 signaling in the endothelium (51, 126).

ApoM-Fc has been characterized to supplement ApoM, demonstrating efficacy in murine models of vascular diseases, including hypertension, myocardial infarction, diabetic nephropathy, and acute models of neovascular AMD (51, 91, 126, 127, 128, 129). Pharmacokinetically, ApoM-Fc can be administered intraperitoneally, subcutaneously, or intravenously in mice. The Fc portion of the fusion protein significantly extends its circulating half-life, protecting it from renal clearance and degradation. A dose of 4 mg/kg was shown to mimic native plasma levels of ApoM-Fc, peaking after 6 h and remaining detectable in plasma for up to 72 h, indicating sustained systemic exposure. Together, these results in a terminal half-life that is approximately four times longer and a clearance rate about three times slower than the native, nonfused protein, consistent with the behavior of other Fc-fusion proteins (104, 130, 131). Crucially, ApoM-Fc, unlike Food and Drug Administration–approved small-molecule S1PR modulators (*e.g.*, FTY720), does not alter circulating lymphocyte counts or induce immunosuppression (51, 132). Similarly, bradycardia is also not induced by ApoM-Fc, presumably because of the lack of activation of G protein–gated inward rectifier channels in the SA node of the heart.

ApoM-Fc was initially recognized for its antihypertensive properties. A single systemic administration reduced systolic blood pressure by approximately 40 mm Hg in angiotensin II-induced hypertensive mice, with effects persisting for up to 192 h after the dose. The underlying mechanism was the S1PR1 dependent, as it enhanced the phosphorylation of endothelial NO synthase and increased plasma nitrite levels (a marker of NO production). The antihypertensive response was further attenuated by S1PR1 antagonist W146, thereby confirming the involvement of S1PR1-dependent mechanisms (133). The same study further outlined ApoM-Fc-mediated cardioprotective effects after MI. ApoM-Fc attenuated ischemia/reperfusion injury in mice, reducing myocardial damage and improving recovery. These results align with and support HDL's established effects of cardioprotection in ischemic injury (51).

ApoM-Fc applications have since expanded to other more chronic vascular pathologies of aging and AMD. In aged mice (>20 months), ApoM-Fc administration attenuated fibrosis and enhanced regeneration in injured lungs and kidneys in an S1PR1-dependent manner (130). This effect highlights the importance of the ApoM–S1P–S1PR1 axis in organ repair during aging and potential applications for chronic vascular syndromes. Vision-threatening diseases of the retina are primarily caused by chronic vascular pathology because of aging or metabolic insults, such as abnormal blood vessel growth, leakage, and occlusion, which leads to retinal ischemia, edema, and ultimately irreversible vision loss (134). Neovascular age–related macular degeneration or “wet” AMD is an advanced, vision-threatening form of AMD in the elderly. In an acute laser choroidal neovascularization model for neovascular age–related macular degeneration, systemic administration of ApoM-Fc did not only effectively attenuate neovascularization, comparable to the standard of care anti–VE growth factor treatment, but also effectively suppressed pathological vascular leakage (104). ApoM-Fc's suppression of pathological retinal neovascularization was further supported by a study investigating systemic administration of ApoM-Fc in the oxygen-induced retinopathy mouse model (127). ApoM-Fc suppressed pathological neovascular tuft formation *via* S1PR1 action. Together, ApoM-Fc counteracted abnormal blood vessel growth in two validated and clinically relevant mouse models of proliferative retinopathies.

Building on the promising vasomodulating properties of ApoM-Fc, efforts have been made to create a second generation of ApoM-fusion protein with further-reaching therapeutic properties and applications. ApoA1-ApoM is a fusion protein of ApoM and Apo-A1, combining the provascular effects of both apolipoproteins. Apo-A1M forms HDL-like nanoparticles ∼10 to 12 nm in size, which bind S1P stably *via* the ApoM lipocalin fold and allow for docking to endothelial scavenger receptors, such as SR-B1 *via* a flexible linker to Apo-A1. Apo-A1M increases barrier function through Gα_i_-biased S1PR1 activation *via* ApoM–S1P and suppresses tumor necrosis factor alpha–induced inflammation and leukocyte activation, LPS induced cytokine storm and neutrophil infiltration in sterile peritonitis models. Apo-A1M additionally blocks thrombin-induced vascular leak as well as platelet aggregation and promotes cholesterol removal *via* ABCA1–ABCG1 transporters in macrophages through Apo-A1. Thus, Apo-A1M is a multifunctional, HDL-mimetic fusion protein with potent endothelial-protective, anti-inflammatory, antithrombotic, and potential antimicrobial properties, primarily through S1PR1 signaling and immune modulation (51).

Alternatives to ApoM-derived biologics for the treatment of cardiovascular and metabolic diseases are next-generation small-molecule S1PR1 agonists. SEW2871 and SAR247799 are promising S1PR1 agonists that act with high specificity and in a Gαi/o-biased manner. In a murine ischemia reperfusion model for myocardial infarction, SEW2871 significantly improved cardiac function and adverse remodeling by reducing apoptosis in uninfarcted myocardium in an S1PR1-dependent manner. SEW2871 also increased reparative macrophage proliferation and suppressed proinflammatory cytokines 50% to 70%, improving ejection fraction post-MI (135, 136, 137). Notably, SEW2871 was reported to cause lymphopenia and cause severe side effects in sepsis because of immunosuppressive effects, presenting major clinical caveats (138, 139, 140). SEW2871 also significantly prolongs ventricular tachycardia and fibrillation during reperfusion in 60% of rat hearts after ischemia–reperfusion injury, leading to irreversible reperfusion arrhythmias (141). To address these caveats, a novel S1PR1 agonist SAR247799 was described. SAR247799 activates S1PR1 G-protein signaling to a greater extent than beta-arrestin, thus minimizing S1PR1 internalization, desensitization, and avoiding lymphopenia. In addition, structural chemical optimization achieved 100-fold selectivity for S1PR1 over S1PR3, minimizing bradycardia risks associated with nonselective agents (142). In pigs with induced coronary endothelial damage, intravenous administration of SAR247799 led to an improvement in microvascular hyperemic response, which is a measure of coronary microvascular function (143). In models of myocardial ischemia and diabetic cardiomyopathy, SAR247799 improved myocardial perfusion and decreased cardiac hypertrophy and fibrosis, by restoring endothelial–cardiomyocyte paracrine signaling and reducing inflammation (144). SAR247799 is the first S1PR1 agonist to enter a randomized controlled trial in type 2 diabetes patients with endothelial dysfunction (NCT03462017). SAR247799 (1 or 5 mg daily for 28 days) improved flow-mediated dilation of the brachial artery, a marker of endothelial health, with efficacy at least comparable to the clinical benchmark sildenafil. Both doses were well tolerated, with minimal-to-no lymphocyte reduction and no significant effect on blood pressure. Small-to-moderate heart rate decreases were observed, suggesting a possible risk for bradycardia (145).

While small-molecule S1PR1 agonists have shown preclinical and clinical promise in alleviating endothelial dysfunction without causing lymphopenia, bradycardia remains a considerable caveat. Biologic modalities, such as ApoM-fusion proteins, have not shown indications of causing bradycardia while retaining vascular protective effects observed in small-molecule agonists.

Biologics, such as ApoM-fusion proteins, may achieve better targeting and signaling bias control for GPCR agonism/antagonism over small molecules. This reduces associated off-target effects of small molecules, which often interact with many other targets, albeit with varying affinities. Moreover, ApoM-bound S1P does not efficiently induce beta-arrestin–mediated receptor internalization, thus allowing more sustained endothelial protective signaling (146). In addition, ApoM fusion proteins of large size restricts tissue penetration, minimizing unintended interactions with intracellular targets compared with small molecules, which can cross membranes more readily, leading to systemic side effects (147, 148). Biologics can further be modified and exhibit remarkable versatility. For example, bispecific antibodies and antibody–drug conjugates have opened novel therapeutic avenues for a diverse range of diseases, which lack small molecules (149). On the other hand, in addition to the high costs associated with manufacturing in comparison to small molecules, biologics present limitations in administration and immune response. First, their administration necessitates injection, which can be challenging compared with oral administration. Second, biologics may trigger immune responses, demanding engineering to mitigate this risk (150). While assumptions can be made that biologics such as ApoM-Fc might present advantages over small-molecule agonists for S1PR1, no comparative study has been reported to date and thus presents a crucial study yet to be performed (Table 3).

**Table 3:** Potential advantages and disadvantages of pharmacological small molecule *versus* biologic S1PR1 agonists

Property	S1PR1 agonist	
	Biologics (*i.e*., ApoM-fusion proteins) (51)	Next-generation small molecules (*i.e*., SAR247799) (143)
Route of administration	Injection (subcutaneous, intraperitoneal, intravenous) (153)	Oral or injection (154)
S1PR selectivity	S1PR1 and S1PR3 (51)	S1PR1 (146)
S1PR1 Gi-biased agonism	+++ (51)	+++ (143)
S1PR1 internalization	MMild internalization (less than FTY-720)—normal effect on the β-arrestin (51)	Minimal internalization—very weak effect on the β-arrestin (143)
Heart rate modulation/bradycardia	-	Small to moderate heart rate decreases in patients (145)
Lymphopenia	-	Minimal reduction of lymphocytes in patients (145)
Vascular tissue specificity	Biologics are compartmentalized to the circulation and closely adjacent tissues (153)	Small molecules can diffuse through cell membranes and spread throughout the body (154)
Immune reaction risk	Potentially—requires engineering (155)	-
Clinical prove of concept	-	Improved flow-mediated dilation of the brachial artery comparable to sildenafil in patients with type 2 diabetes (145)

## Conclusions, questions, and future directions

**Figure 5:**
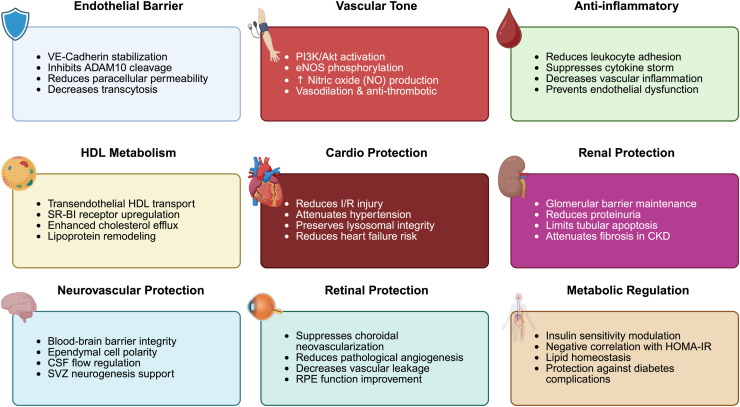
**Summary of proposed biological functions of ApoM.** ApoM, apolipoprotein M.

This review underscores the pivotal role of ApoM in vascular biology, primarily through its function in chaperoning S1P in circulation and its modulation of endothelial S1PR1 signaling. Functions of ApoM–S1P encompass endothelial barrier preservation, anti-inflammatory signaling, and HDL transcytosis, which are essential processes for maintaining vascular health in both physiological and pathological conditions (Fig. 5). The accumulating body of preclinical and clinical evidence positions ApoM as a promising biomarker for precision medicine and therapeutic target in cardiometabolic, neurovascular, and inflammatory diseases. Despite recent advances, critical questions remain to be answered.

The role of ApoM in transporting S1P with highly specific and favorable endothelial S1PR1 binding kinetics has been extensively documented. However, the exact mechanism of S1P release from ApoM to S1PR1 on the endothelium remains elusive. Furthermore, post-translational modifications and interactions with other circulating proteins may influence ApoM–S1P interactions on HDL or in relation to S1PR1 activation, potentially affecting its binding, signaling, or clearance characteristics and require further investigation.

Although the liver and kidneys are the primary sites of ApoM synthesis, the tissue-specific roles and regulation of ApoM, particularly in adipose, enteric, neural, and retinal tissues, remain poorly understood. It is imperative to identify the consequences of local ApoM deficiency in these compartments. Furthermore, while the contribution of ApoM from adipose tissue is minimal, the regulation and potential differences in liver or kidney-derived ApoM must be characterized, particularly because of the significant role of ApoM in lipid metabolism.

Correlative data suggest strong associations between ApoM levels and disease severity in CKD, diabetes, ocular, and cardiovascular disease. Prospective studies are needed to validate its predictive utility and integrate it into clinical decision-making algorithms. Moreover, the potential redundancy and compensation provided by other chaperones, such as ApoA4 or albumin, in ApoM-deficient states necessitate further investigation. It is imperative to elucidate the conditions under which these alternative carriers can compensate and the physiological consequences they entail in both physiology and pathophysiology such as MODY patients with undetectable ApoM levels.

Estrogen receptor–mediated transcription of ApoM and its modulation by HNF-4α, FOXOs, LRH-1, and liver X receptor indicate complex endocrine control of S1P chaperones. Investigations into sex differences have largely been neglected in animal and clinical research, making it imperative to include such in future studies and how such can influence in ApoM-linked diseases onset, severity, or therapeutic responses.

Conflicting clinical observations, such as increased ApoM in some inflammatory bowel diseases and decreased levels in sepsis or SLE, suggest context-dependent regulatory mechanisms. These differences could be attributed to alterations in transcriptional regulation, post-translational modifications, or altered clearance and require further investigation.

While ApoM fusion proteins (ApoM-Fc and ApoA1-ApoM) exhibit efficacy in preclinical models, clinical translation necessitates meticulous evaluation of immunogenicity, off-target effects, and the optimal balance between prosurvival and proliferative S1PR1 signaling pathways. A notable advantage of ApoM-Fc is its capacity to circumvent lymphopenia and bradycardia. Nevertheless, further research is required to ascertain whether this effect persists in chronic dosing or under inflammatory conditions, such as diabetes mellitus. Given the antagonistic effects of S1PR2 on vascular integrity, it is intriguing to explore whether a combinatorial approach of S1PR1 agonism and S1PR2 antagonism may yield synergistic benefits, particularly in pathologies, such as stroke, pulmonary edema, or sepsis.

**Figure 6:**
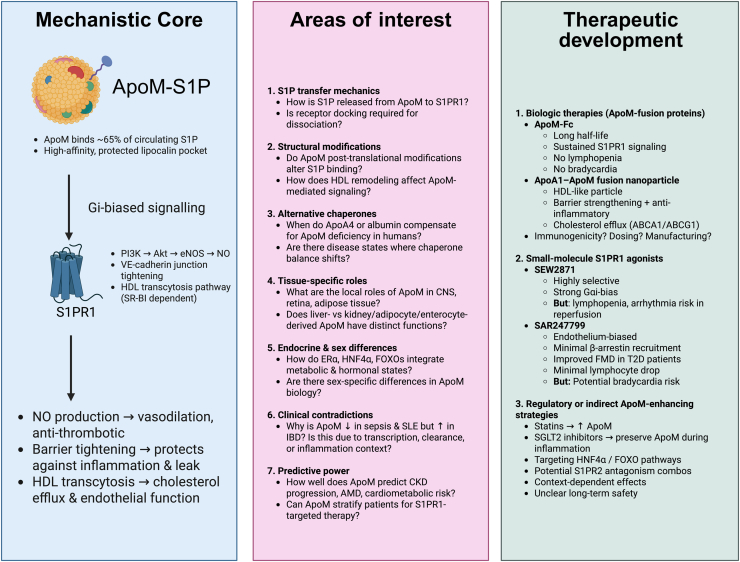
**Mechanistic core, areas of interest, and current state of therapeutic development of ApoM.** ApoM, apolipoprotein M.

Finally, while ApoM–S1P signaling has emerged as a central node in vascular health, bridging lipid metabolism, inflammation, and endothelial function, translational gaps persist. Addressing these questions will be crucial for advancing ApoM-targeted therapeutics from bench to bedside and for refining our understanding of endothelial homeostasis in health and disease (Fig. 6).

## Declaration of generative AI and AI-assisted technologies in the writing process

Generative AI (Sonnet 4.5) was used to visualize Figure 4.

## Conflict of interest

T. F. D. declares no conflicts of interest with the contents of this article. T. H. is listed as an inventor on patent applications on ApoM fusion proteins.
